# How to bridge the nurse innovation–diffusion gap? An in-depth case study of Create4Care

**DOI:** 10.3389/fpubh.2023.1209965

**Published:** 2023-08-03

**Authors:** Coen Rigtering, Lara J. Spaans, Jeroen P. J. de Jong

**Affiliations:** Entrepreneurship Section, Utrecht University School of Economics, Utrecht University, Utrecht, Netherlands

**Keywords:** single case study, ecosystems, nurse, innovation diffusion, practice guidelines

## Abstract

**Introduction:**

Nurses frequently innovate in response to operational failures, regulations, procedures, and/or other workflow barriers that prevent them from delivering high-quality patient care. Unfortunately, most nurse innovations do not diffuse to a broader audience, depriving other nurses from taking advantage of solutions that have already been developed elsewhere. This under-diffusion is problematic from a societal and welfare point of view. The goal of this paper is to understand how diffusion shortage of nurse innovations can be reduced.

**Methods:**

We develop a qualitative case study of a medical makerspace at the largest academic hospital in the Netherlands. This medical makerspace reported unusually high rates of nurse innovation diffusion. Our data collection includes on-site observations, archival data, secondary data, and fifteen in-depth interviews with key informants. Qualitative coding procedures and a combination of deductive and inductive reasoning are used to analyze the data.

**Results:**

Our data show that personal, organizational, regulatory, and market barriers prevent nurses from further developing and diffusion their innovations in an anticipatory manner. That is, because nurses expect that transforming an initial solution into an innovation that can be shared with others will be too time consuming and difficult they do not proceed with the further development. The medical makerspace that we investigated adequately addresses this problem by developing an innovation ecosystem that largely takes over the innovation and diffusion process.

**Discussion:**

We provide a concrete example of how a medical makerspace, and innovation support systems in a broader sense, can be designed to more adequately address the nurse innovation-diffusion gap. The two main elements of the practical solution that we identified are: (1) Support systems should facilitate that others may lead the development and diffusion of innovations and (2) The support system should promote that actors integrate their functional specializations within an innovation ecosystem. We make two theoretical contributions. First, we contribute to understanding barriers in the nurse innovation-diffusion process from a psychological point. Second, we identified that an ecosystem perspective is beneficial to develop innovation support systems in which diffusion occurs more often.

## 1. Introduction

Promoting nurse innovation has become an important goal in healthcare systems around the globe (1–3). In the United States, for example, the National Academy of Medicine stressed the importance of promoting an innovative mindset among nurses in their Future of Nursing 2020–2030 report (4). Successful nurse innovation is often associated with improved medical service quality, improved effectiveness of treatments, higher levels of job satisfaction, improved healthcare access, and simplified processes in delivering healthcare services (1, 5). Because nurses make up the largest segment of the healthcare workforce (6) and provide up to 80% of primary healthcare (7), nurse innovation is broadly recognized as a solution to combat the rapidly increasing healthcare costs, national nursing shortages, and variations in healthcare quality (8).

The American Nurses Association defines nurse innovation as actively seeking and developing new methods, new technologies, and new tools to promote health, prevent diseases, improve the quality of care of patients, and the application of these innovations via teamwork and support channels (9). Nurses have a long history of being innovative (10) but, ironically, are not always recognized as innovators. Nurses commonly find solutions to operational failures, regulations, procedures, and/or other workflow barriers that prevent them from delivering high-quality and safe patient care. Unlike physician innovators, they seldom develop technologically advanced innovations based on systematic research and development (8, 11–13). Unfortunately, the vast majority of the useful solutions that nurses develop do not result in systematic improvements in the healthcare system. Nurses typically do not commercialize their innovations and/or do not share them with nurses working at other departments or other hospitals—that is, their innovations tend not to diffuse (3, 8, 13). While non-diffusion is a general problem within health care (11, 14), it applies to nurse innovations in particular. For example, studies show that patent applications by nurses are very rare (15, 16) and that it takes a staggering 15 years before an evidence-based nursing practice is broadly adopted (17). Non-diffusion is problematic from a societal and welfare point of view as nurse innovations generally have a high return on investment (3) and non-diffusion prevent patients from benefitting from healthcare improvements that are developed elsewhere (8, 12, 13, 18).

Various reasons for the lack of diffusion among nurse innovations have been identified. For example, scholars have pointed out that nurses commonly lack innovation abilities (1), technological abilities (19, 20), and knowledge about the innovation process (13, 21). Other scholars have identified that hospitals generally lack a culture and/or infrastructure that supports innovation (2, 8, 11, 22). Commonly proposed solutions to combat these issues are to make innovation or entrepreneurship a key element of the nursing curriculum (1, 21, 23, 24) to appoint nurse innovation leaders (13), or to set up Nursing Innovation Centers that bring together faculty and students [see (22)]. Yet, despite implementing these solutions, innovation rates remain rather low (2, 8).

Recently, scholars and practitioners have been building on the principles of open innovation (25–27) to propose that innovation support systems such as fablabs (28), living labs (29), and medical makerspaces (3, 30, 31) are a key tool to increase nurse innovation. Medical fablabs, living labs, and makerspaces offer (staffed) innovation assistance facilities with access to prototyping equipment such as 3D printers and laser cutters (3, 28, 30). Although these facilities do enable nurse innovation, the diffusion of the innovations remains a persistent problem. For example, Svenson and Hartmann (3) conclude that medical makerspaces encourage nurse innovation and provide potential returns of up to 14 times the investment needed to establish and run the makerspaces. Yet, a very limited amount of this potential is realized, owning to the under-diffusion of the innovations (3).

The purpose of this article is to better understand why the nurse innovation–diffusion gap is so persistent and how this gap can be overcome. Although significant progress has been made in understanding the importance of innovation support systems, studies mainly describe the process of setting up an innovation support system (31), the general design features (22), or outcomes (3). A more *in-depth* understanding of why innovation support systems oftentimes fail to generate high levels of nurse innovation diffusion is still missing. The research questions for this paper are:

**Research Question 1:** What prevents nurses from diffusing their innovations?

**Research Question 2:** How can innovation support systems be designed to adequately address these diffusion barriers?

## 2. Methods

### 2.1. Qualitative approach

We conducted a qualitative case study (32) of nurse innovations developed at Create4Care, the medical makerspace of Erasmus Medical Center. A case study was used to understand the complex phenomena of nurse innovation and diffusion in its natural, organizational setting. The case study was developed over two distinct phases. During Phase 1, one of the authors spent several months at Create4Care as an embedded researcher. She had full access to all internal databases of Create4Care, observed the various professionals, and had regular talks to better understand the context and diffusion processes. This first phase was mainly for exploratory purposes and the insights that we developed provided input for our subsequent data collection (Phase 2) in which we conducted in-depth interviews and collected secondary data.

### 2.2. Context

Erasmus Medical Center is the largest university medical center in the Netherlands. In 2021, Erasmus Medical Center had an annual turnover of € 2.1 billion, 659,317 outpatient visits, and 30,771 patients were admitted. The organization has 16,180 employees (including subsidiaries) and 4,093 students. Its three core tasks are patient care, education, and research.

Create4Care is the medical makerspace department of Erasmus Medical Center. Create4Care directly reports to the board of directors and is run by a professional manager (0.5 FTE). The department was set up by a nurse who identified that many colleagues struggled while developing and diffusing nurse innovations. In practice, the department is very informally organized and the nurse that set up the department co-manages the medical makerspace with the aforementioned professional manager.

The case was brought to our attention as Create4Care reported unusually high rates of nurse innovation diffusion. To confirm if Create4Care indeed provided a best practice in terms of the diffusion of nurse innovations and was a suitable setting for our study [see (32)], our embedded researcher developed a database of all finished nurse innovation projects (26 finished projects and 19 ongoing projects at the time of the data collection). She documented all diffusion efforts done, and the relevant diffusion pathways [commercial or peer-to-peer, see (33)]. In 24 out of the 26 nurse innovations, a substantial effort had been made to diffuse the innovation. Twelve of the 24 innovations had actually diffused; four were in the process of being introduced to the market by producers (commercial diffusion) while eight were directly adopted by peers working at Erasmus Medical Center and other hospitals in the Netherlands (peer-to-peer diffusion). After understanding the context and verifying the unusually high rates of nurse innovation diffusion,^1^ we proceeded with Phase 2 (interviews and secondary sources).

**Figure 1:**
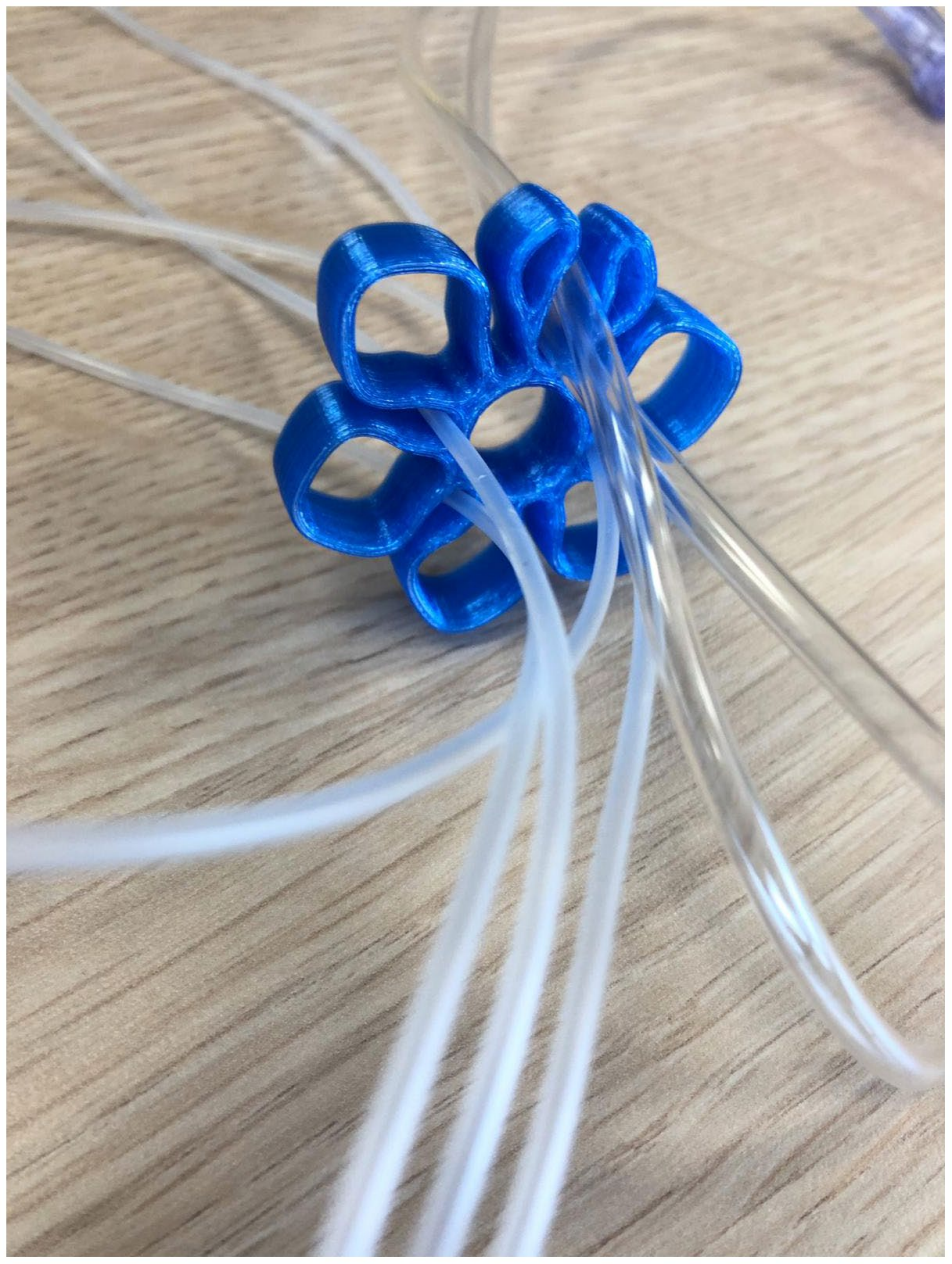
The infusion lines flower developed at Create4Care.

An example of an innovation developed at Create4Care is the Infusion Lines Flower (see Figure 1). In early 2018, a nurse in the Children’s Intensive Care unit noted that the spaghetti of infusion lines surrounding hospital beds could create safety hazards as lines can get mixed up after moving patients. She, furthermore, noted that the organization of infusion lines was very time-consuming. After recognizing this problem, the development of a solution started in September 2018. In 2019, the first 3D-printed prototype was ready for testing at the Children’s Intensive Care unit. A version ready for mass production followed in early 2020. The invention was later that year adopted by a commercial producer and by the end of 2020 the product was introduced in the Dutch, German, and Scandinavian markets.

### 2.3. Sampling and reliability

The embedded researcher identified 13 key actors [see (34)] who took charge of innovation development and diffusion, based on her on-site observations and informal discussions. Each of these key actors was invited for an interview (and accepted our invitation). We also scheduled two additional interviews with the founder of Create4Care to learn more about the broader context. The interviews were conducted over a two-month interview period. Theoretical saturation (32) was achieved after approximately 12 interviews, meaning that no additional insights that are of theoretical importance were obtained in the last three interviews.

The average interview time was about 75 min with interviews typically lasting between 60 and 90 min. To ensure reliability, we collected secondary data at Erasmus Medical Center/Create4Care (for example, annual reports and strategy reports), consulted external sources (practitioner magazines and newspaper articles), and used the field notes that were collected by the embedded researcher in Phase 1 to triangulate the interview data. Tables 1, 2 provide an overview of the data collected during Phases 1 and 2.

**Table 1:** Overview of primary data collection.

		Phase 1				
		*Exploratory data collection*				
	Data collected by the embedded researcher	Data on the diffusion of finished nurse innovation projects, field notes, and on-site observations.				
		**Phase 2**				
		***Interviews with key informants identified by the embedded researcher in Phase 1***				
**Respondent #**	**Job title**	**Organization/department**	**Years of working experience**	**Educational level**	**Type of diffusion effort**	**# of interviews**
1	Senior business development manager	Erasmus Medical Center/Technology Transfer Office	4	Master	Creating a suitable environment for diffusion/diffusing individual innovations	1
2	Junior business development manager	Erasmus Medical Center/Technology Transfer Office	1	Master	Diffusing individual innovations	1
3	Coordinator	Erasmus Medical Center/Create4Care	30	Ph.D.	Creating a suitable environment for diffusion/diffusing individual innovations	3
4	Manager	Erasmus Medical Center/Create4Care	7	Master	Creating a suitable environment for diffusion/diffusing individual innovations	1
5	Electronic engineer	Erasmus Medical Center/Create4Care	1	Master	Diffusing individual innovations	1
6	Instrument maker	Erasmus Medical Center/Medical Instruments Department	16	Bachelor	Diffusing individual innovations	1
7	Business advisor	Erasmus Medical Center/Medical Instruments Department	16	Bachelor	Creating a suitable environment for diffusion/diffusing individual innovations	1
8	Quality advisor/nurse	Erasmus Medical Center/Children Intensive Care	18	Bachelor	Creating a suitable environment for diffusion/diffusing individual innovations	1
9	Nurse	Erasmus Medical Center/Center for Home Ventilation and Respiratory Disorders in Children	27	Vocational training	Diffusing individual innovations	1
10	Technical coach	Rotterdam University of Applied Sciences	15	Master	Creating a suitable environment for diffusion/Diffusing individual innovations	1
11	Technical coach	Rotterdam University of Applied Sciences	24	Master	Diffusing individual innovations	1
12	Technical coach	Rotterdam University of Applied Sciences	22	Master	Diffusing individual innovations	1
13	Entrepreneur	BestCare Solutions	21	Master	Diffusing individual innovations	1

**Table 2:** Overview secondary data sources.

	Secondary data sources	
Type of data source	Coverage in years	Total number of documents
Hospital strategy plan 2019–2023	2019–2023	1
Annual report Erasmus Medical Center	2018–2020	3
Hospital online blog	2020–2021	4
		1
Newspaper articles	2020–2021	3
Create4Care page on the Website of Rotterdam University of Applied Sciences	2020–2021	1
Practitioner magazines	2016–2021	4

### 2.4. Data collection instruments and units of analysis

We developed a semi-structured interview protocol designed to identify the main barriers that prevent nurse innovations from developing and spreading their solutions to a broader audience, and the procedures used within Create4Care to overcome these barriers. Additionally, we asked respondents to reflect on their role within the process and their motives to participate in Create4Care. Wherever possible, we asked the interviewees to provide concrete examples of barriers, procedures, actions, roles, and motivations. For example, to better understand the procedures within Create4Care, we first identified specific nurse innovations to which the interviewee had contributed. We asked the interviewee to provide examples of concrete actions that helped to develop and diffuse the nurse innovation(s). This approach, which prompts interviewees to provide specific examples of events, forces interviewees to use episodic memories and significantly increases the accuracy of the obtained information (35, 36). After eliciting these specific events, we asked more open follow-up questions [for example, What elements were crucial? How did you do this? Why was the approach (un)successful? Why is this procedure being used?] to gain a deeper understanding of the innovation and diffusion procedures. Most interviews were conducted in person, on location, and in a private room. Because of COVID restrictions, a limited number of interviews were held online. We recorded each interview. The interview protocol did not change throughout the event of the study. The interview protocol can be found in Supplementary material A.

### 2.5. Data processing and ethics

We obtained approval for the research from the Ethical Committee of the Faculty of Law, Economics, and Governance (nr. 2020–019) of Utrecht University. All interviewees were informed of the data protection and processing procedures before the start of the interview. They verbally provided consent for using their anonymized data for research purposes. The interviews were fully transcribed for further data analysis. The interview data was stored on the secure servers of Utrecht University. We used randomly generated numbers to anonymously store the interview transcripts. The interviews are displayed in random order in Table 1. In the text, we use #1 to refer to Interviewee #1 in Table 1 and #2 to refer to Interviewee #2 in Table 1.

### 2.6. Data analysis and rigor

We followed procedures recommended by Gioia et al. (37) for systematically analyzing qualitative data and achieving qualitative rigor. The approach of Gioia et al. (37) consists of three stages: open coding, axial coding, and selective coding. Open coding involves generating categories (also known as second-order codes) that are derived from interview transcripts, secondary data sources, and field notes. These are then linked to the categories to classify meaningful pieces of information. During axial coding, the categories are arranged into more abstract theoretical dimensions in a meaningful way by linking categories with each other and creating a hierarchical order. Finally, during selective coding, categories are organized around core explanatory concepts to build the theory (38).

**Figure 2:**
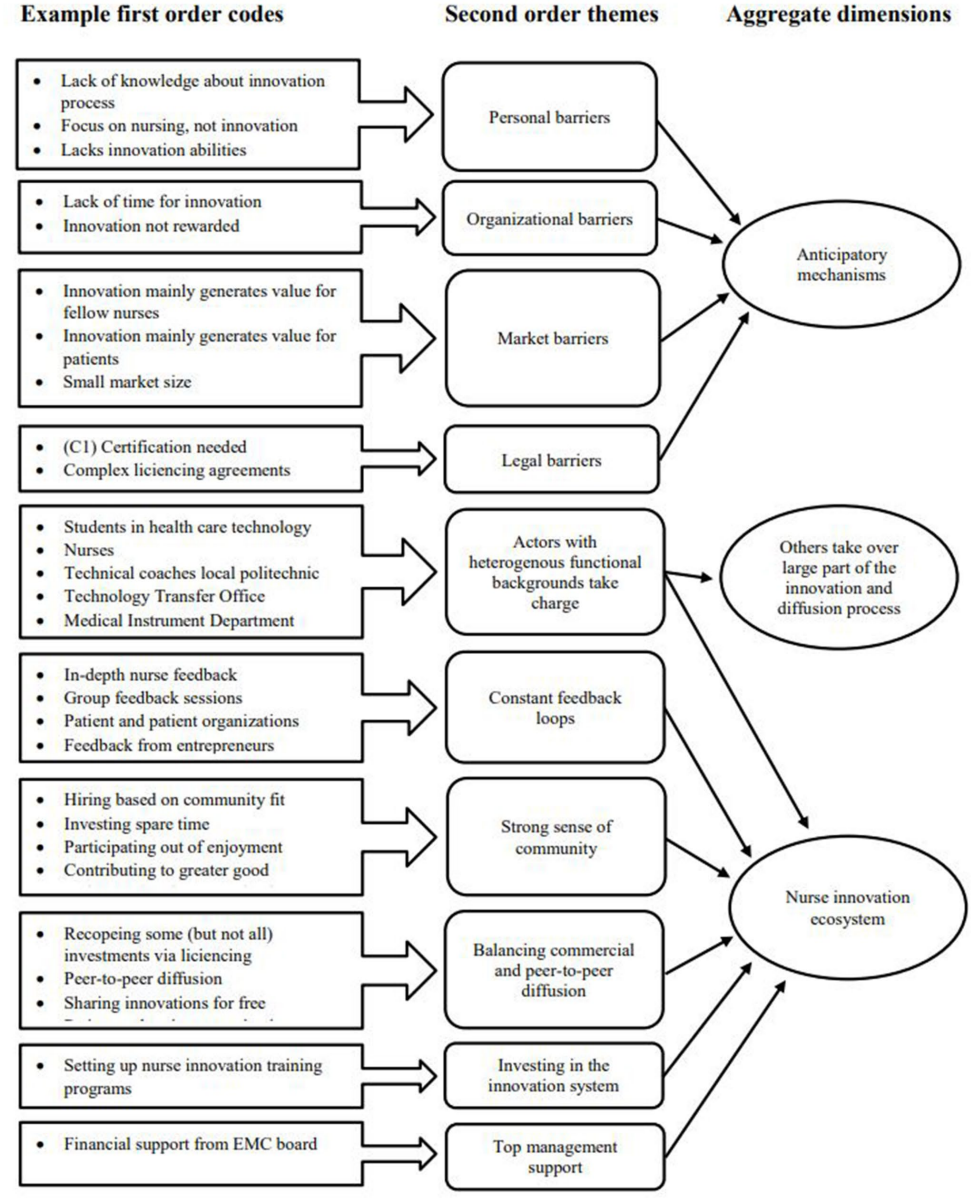
Final coding scheme.

To facilitate the coding process and knowledge sharing among the authoring team, we organized numerous discussion sessions. These discussion sessions took place directly after a set of interviews. The interview(ers) took the lead in describing the main insights that were obtained during the interview(s) to the other researchers. This ensured that all researchers were up to date with recent developments and helped in creating a shared understanding of the Create4Care case. After the interviews were completed, we continued with these discussion sessions but switched to formal coding of the qualitative data where we made use of a combination of interview transcripts, secondary data, and field notes collected by the embedded researcher to triangulate the data. During all sessions, we relied on a combination of deductive and inductive reasoning [see (39, 40)] to situate our findings within existing work on nurse innovation and innovation diffusion. We used deduction to sort and structure the data according to the main components of the proposed framework (41). Induction was used to uncover unexpected findings and deepen the theoretical analysis of the data (37, 40, 41). Figure 2 provides an overview of the outcome of the coding process. During the axial coding, we created 10 s order themes that related to three aggregate dimensions. The first aggregate dimension (Anticipatory mechanisms) describes the way innovation barriers affect the diffusion of nurse innovation. The second (Others take over a large part of the innovation and diffusion process) as well as the third aggregate dimension (Nurse innovation ecosystem) capture how the nurse innovation–diffusion gap is bridged at Create4Care.

## 3. Results

### 3.1. The nurse innovation—diffusion process: barriers that prevent diffusion

A full innovation and diffusion sequence consist of three phases (i) problem identification and prototyping to fix the problem, (ii) continued development, and (iii) diffusion (42). Continued development may include design, technological development, market research (to check for market potential and if there are similar solutions that are already available), certification, and business model development. Diffusion includes setting up production, distribution, and sales in the case of commercial production. In peer-to-peer diffusion, it is a less demanding task but still includes sharing design files with instructions in a format that other people can understand (43). During our interviewees, a large variety of barriers were mentioned that prevent nurse innovations from spreading to a broader audience. Based on previous work (1, 3, 11, 22), we grouped these reasons into personal, organizational, regulatory, and market barriers (see Figure 2). Personal barriers capture the innovation and technical skills needed to develop an innovation, as well as a nurse’s belief in those innovation and technical skills (44). Organizational barriers capture hospital-specific structures that prevent the development of nurse innovations. Think of barriers such as insufficient time to develop innovations or bureaucratic procedures that stiffen further development (11). The complex certification processes that are needed for introducing medical innovations, and medical devices that require Class I or higher certification, in particular,^2^ create regulatory barriers for nurse innovators. As documented in the literature [see (3)] and noted by one of our interviewees, “…*meeting all regulatory requirements is a very time-consuming and expensive process*” (#12).

While personal, organizational, and regulatory barriers have been discussed in previous nursing innovation studies (2, 8, 11, 19, 20), market barriers have received little attention. Market barriers relate to the expected market size and the ability to protect nurse innovations via patents. Sufficient market size and protectability are key conditions for commercial diffusion because businesses/investors want to see the market potential and a viable business model to recoup their investments (16, 45). Unfortunately, patents are often not an option because of the low-tech nature of a lot of nurse innovations (# 1, 2). Nurse innovations mainly address unfulfilled user or patient needs and are developed in response to practical problems that nurses experience on the job instead of emerging technology. The highly practical nature of nurse innovations is well-captured by the following quote by one of the nurses (#8) “*initial solutions are sometimes put together with duct tape*.” It is also described in Debono et al. (12) and O’Harra et al. (8). Almost all interviewees furthermore mentioned that a considerable percentage of the innovations that were being developed at Create4Care was not commercially viable because of “*insufficient market size*.” The interview extract below provides a good example. In this example, the innovation addresses a specific problem that nurses of the Children Intensive Care Unit experience. The market potential is very limited, but the innovation does offer substantial value to the nurses themselves.

*“Children that receive heart surgery at the Children Intensive Care unit often have a chest drain, which are very large tubes around their heart area. These tubes run down to a vacuum pump to drain any moisture that occurs during or after surgery. The reservoir that collects the moisture needs to be placed on the floor under the patient’s bed. To check the amount of moisture, a nurse needs to read the display on the reservoir. During the evening and night shifts, we need to do this every hour to check if there is any bleeding or alike. We could lift the reservoir, to check the display. However, because of the large tubes, the kids would immediately notice this and it can be quite painful for them. … So, every hour one of the nurses needs to get on their knees to check the display. Not only the colleagues who are 25 years old but also our colleagues of 60 years and older who are already frequently struggling with back issues. We do not have one of these patients, but usually 2 or more simultaneously. … The solution that was developed is a separate remote display, which is very helpful, especially for our older colleagues”* (#8).

If there is insufficient market size, peer-to-peer diffusion is the only way a nurse innovation can still diffuse. Yet, peer-to-peer diffusion is particularly complex in the medical sector. Regulations do not only apply to commercial producers but “*other hospitals are also not able to adopt an innovation without certification being complete*” (#1). This implies that approximately the same effort and investment are needed for peer-to-peer diffusion as for the development of commercial innovations without any means to recoup these investments.

**Figure 3:**
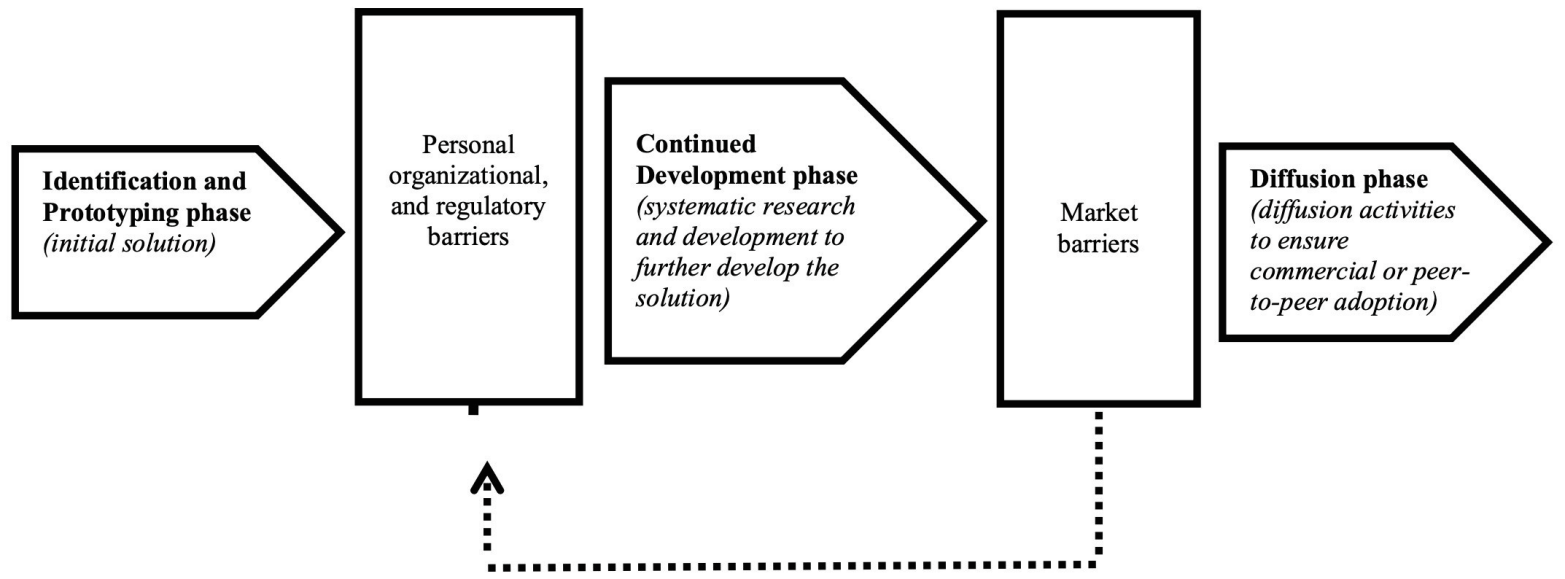
Barriers to nurse innovation diffusion.

In Figure 3, we position the different barriers in the nurse innovation–diffusion process. Previous research suggests that innovation education (8, 13), technical expertise (19, 20), and innovation support systems (2, 3) would significantly lower the barriers for developing and diffusing a nurse innovation. Yet, even when nurses possess the right expertise and are provided with sufficient support, developing a viable solution may still be “*a bridge to far*” (# 7, 8). Intentional actions, such as deciding to develop a solution or innovation, are regulated by forethought; individuals form beliefs about what they can and cannot do, set goals, and plan courses of action that are likely to produce desired outcomes (46). Nurses thus need to assess their ability to act, need to determine their aspiration level, how they will proceed, and need to assess the likelihood success (47). These assessments are relative to the opportunity that the nurse has identified *and* any other goals that they might have [see (47, 48)]. In other words, innovation competes with the professional and personal goals of the nurses. Given these competing goals, the long and complex development trajectories of most nurse innovations significantly reduce the likelihood that a nurse foresees beneficial outcomes and decides to proceed. We thus posit that these barriers do not only create objective constraints, they also function as anticipatory mechanisms. That is, because nurses anticipate that personal, organizational, and regulatory barriers reduce the likelihood of success, they do not proceed with the further development. In addition, if it is clear *a priori* that market potential might be limited, market barriers also affect the likelihood that nurses continue with the development in an anticipatory manner (this secondary effect is visualized by the dotted lines in Figure 3).

### 3.2. Overcoming the nurse innovation—diffusion gap: the Create4Care approach

**Figure 4:**
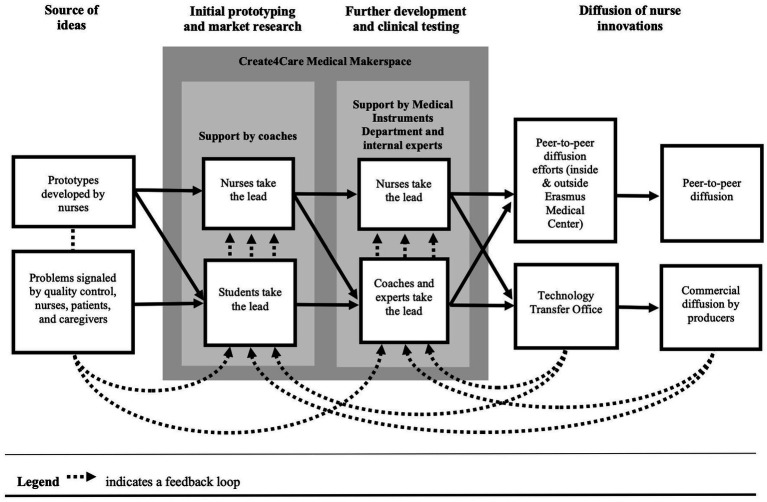
Overview of the innovation and diffusion processes within Create4Care.

After developing a more in-depth understanding of the nurse innovation–diffusion gap, we focus on how Create4Care manages to overcome this gap. Figure 4 provides a stylized overview of the innovation and diffusion processes used within Create4Care. We discuss the approach in more detail below.

#### 3.2.1. Actors with heterogenous functional backgrounds take charge of innovation and diffusion

A nurse innovation project at Create4Care can start in various ways. A nurse innovator may reach out to Create4Care when he or she has developed an initial prototype. The nurse innovator can then make use of the makerspace facilities (which include prototyping equipment such as 3D printing devices and laser cutters) to develop a more professional prototype and support from various professionals (technical, commercial, and legal support). This route is very similar to that of other medical makerspaces (3, 30, 31). In practice, however, it is seldom used. Instead, in most projects, Create4Care takes the lead in the (further) development of a prototype. Many of these projects are still initiated by nurse innovators who showcase prototypes to Create4Care but nurses, patients, and caregivers can also come to Create4Care with ideas for improvements or problems that they experience. In addition, Create4Care actively searches for ideas and solutions. One of the Quality Advisors/Nurses (#8) explains how Create4Care is involved in the quality control procedures:

“*All misses or near misses related to patient safety, technical failures,* etc. *are reported in our quality system. Before Create4Care, we then discussed among ourselves [the nurses and Quality Advisors] how we could solve these issues. Nowadays we take a more systematic approach in which we, with the help of Create4Care, first search for existing solutions that are already available on the market. If we cannot find a suitable solution, Create4Care can sometimes help to develop a solution.*”

The coordinator of Create4Care (#3) explains how Create4Care promotes that nurses and patients also proactively voice potential problems and/or solutions.

“*My colleagues and I frequently present our innovations internally and promote Create4Care. Through these presentations, nurses learn about our work and what we can do for them. Increasingly, nurses and patients manage to find us. We carefully listen to their ideas and suggestions and select the ones that we believe we can develop a solution for*.”

Combining the active search with the input via the quality control procedures ensures that there is a constant inflow of new ideas and innovations.

In developing the initial prototype or further developing an existing prototype, Create4Care relies to a large extent on students from a local polytechnic (Rotterdam University of Applied Sciences). These students work on innovations as part of their healthcare technology minor and are supervised by three coaches of the polytechnic. Approximately 70 students participate on an annual basis. This not only ensures that development costs remain rather low, but students also bring new perspectives and ideas.

“*We [nurses] have a fairly limited view on the type of solutions that can be implemented and oftentimes lack technical expertise. Students with a background in healthcare technologies develop totally different ideas and make use of different types of technologies and materials. This allows us to develop better solutions for our patients and colleagues*” (#8).

In the later stages of the development process, the coaches may also contribute to the further development themselves and ensure a certain level of ‘professionalism’. “*An innovation is never finished when the students are ready to graduate. …They sometimes also lack the skills and expertise to make that final step. We then step in to make that final step”* (#10). Experts are then also brought along. For example, an instrument maker (#6) from the Medical Instruments Department can assist in developing a prototype that meets “*the requirements for clinical testing*” (#6) and Create4Care has a part-time electronic engineer (#5) for projects that include more complex hard- and software applications.

Relying on students and professionals for the further development of the innovations does not mean that the prospective users (nurses, patients, caregivers) of the innovation are not involved in the development processes. Each new prototype is discussed with the users for feedback and their input is integrated into each iteration. Feedback is provided informally, but there are also more formal sessions. These sessions vary from in-depth sessions with quality advisors or nurses to shorter group sessions. “*We have regular professional development sessions with large groups of nurses being present. We use some of these sessions to discuss the innovations that are being developed at Create4Care and provide feedback*” (#8). When an innovation is targeted toward patients or caregivers, Create4Care develops the innovations in collaboration with patients/caregivers and/or patient organizations. These feedback loops are visualized at the bottom of Figure 4 as moving from the nurses, patients, and caregivers, to the students, nurses, and professionals that develop the innovations. Through their feedback, nurses and patients mainly contribute to the prototyping and clinical testing phases (see Figure 4). In addition, in the case of peer-to-peer diffusion, they are often the first adopters and serve as innovation ambassadors.

Strikingly, market and regulatory diffusion barriers are addressed already *early* in the development process by involving the Technology Transfer Office and Medical Instruments Department:

“*Each project starts with basic market research to check if there are similar solutions available and to determine if there is market potential”* (#12)*. “Partially this is to check if there are any liabilities or risks that we need to take into consideration”* (#1) “*I often have discussions very early in the development process with the students and coaches about how they can ensure that the innovation that they develop will meet certain standards. Can the innovation be designed in such a way that it meets hygiene standards, that it can be easily cleaned, and that it can be easily produced? This prevents problems and delays later in the development process* “(#6).

In addition, feedback is provided by commercial parties that have already adopted some of the nurse innovations developed at Create4Care. *“I sometimes provide feedback on the innovations that are being developed. Some of these innovations are not interesting for me or my company, but I find it important to still help in developing these innovations and to provide feedback from a more commercial perspective”* (#13). In some cases, this early involvement directly enhances the likelihood of diffusion. “*As part of our marketing research, we are sometimes able to identify partners that might want to sponsor some of the development costs or we can set up a joint partnership with a commercial producer*” (#1).

Commercial diffusion is not the end goal for all innovations, but it is “… *an important way to recoup some of the upfront development costs, which can be substantial*. *Most commonly, we work with licenses*” (#1) Create4Care has set up several channels (including social media channels, networks, and websites) to find commercial parties that are interested in adopting the innovations. “*The advantage of not being able to patent many of the innovations is that we can more easily share innovations. We are able to attract considerable attention for our innovations via social media channels*” (#2).

To mobilize peer demand (within and outside Erasmus Medical Center) for innovations that cannot be commercially diffused, members of Create4Care present innovations during seminars and submit articles to newsletters and alike. Another way is to freely share innovations. For example, “*For the Ampule Breaker project, we distributed copies to other nurses*” (#10). Another option is to freely share innovations with the outside world. “*If there is demand at the (inter)national level but it is too small for commercialization, we just share the CAD or design files with other hospitals and tell them ‘produce it yourself.’*” (#12).

#### 3.2.2. Critical success factors

In summary, Create4Care has two unique features compared to other medical makerspace programs (3, 30, 31). First, Create4Care does not exclusively rely on nurses for the further development of innovations and even actively searches for problems that nurses experience on the job. Second, despite the significant development costs of some nurse innovations, peer-to-peer diffusion is actively promoted. We identified five critical success factors that support this approach: an innovation ecosystem approach, community, ecosystem co-evolution, balancing profit and non-profit, and top-management support.

##### 3.2.2.1. Innovation ecosystem approach

Create4Care brings together a variety of internal (nurses, quality advisors, and technological and legal experts) and external (patients, parents/caregivers, patient organizations, educational institutions, and producers) actors. Each of these stakeholders provides crucial input, sometimes voluntarily (that is, without receiving direct compensation), and works together within an innovation ecosystem. We use the term ‘ecosystem’ to emphasize that innovation and innovation diffusion is enabled through complex interactions between actors and between actors and their physical environment (the makerspace facilities) in a community setting [see (49)]. Cooperation is the result of actors achieving complementary benefits by integrating their functional specializations. Think of nurses who benefit in the long run from providing the students that work on the innovations with detailed feedback or legal specialists who are willing to provide feedback in the early phases of the innovation process to prevent “*costly losses and problems later on*” (#2). The makerspace facilities facilitate informal interactions by providing a physical place where actors can meet, exchange ideas, and can work together on innovations. The coordinator (#3) and manager (#4) of Create4Care act as ecosystem leaders (50) in that they actively promote frequent interactions, institutionalize them via working procedures/best practices, and expand the ecosystem by inviting new members to contribute and benefit from the innovations developed at Create4Care.

##### 3.2.2.2. Community

In organizations, a recognizable community emerges when the population develops an identifiable cohesion that derives from mutualistic interdependence among actors with complementary differences [see (51)]. Despite the large differences between actors in terms of their functional specialization, there is a strong cohesion and, almost without exception, interviewees indicate that advancing the nursing profession and/or helping patients is a decisive factor in why they are motivated to contribute to Create4Care. The strong motivations of the actors to contribute to a common cause result in additional effort (for example, “*I invest substantially more time than formally required*” #1) but also help to overcome arguments that might arise as a result of interdependencies and conflicting interests. The coordinator (#3) explains that safeguarding the community is a key goal. *“We just received an additional budget to hire new people. Yet, we do not hire people simply because we have the budget for it, even if they have the right expertise. New people need to fit in the community.”*

##### 3.2.2.3. Ecosystem co-evolution

Importantly, Create4Care was *not* planned for as a result of corporate policies. Rather, it was established bottom-up by a nurse innovator (currently the coordinator of Create4Care) who worked at the Children’s Intensive Care unit of Erasmus Medical Center. Nurses at the Children’s Intensive Care unit commonly have to rely on workarounds to perform their day-to-day duties as *“providers of medical equipment do not always offer suitable solutions for kids”* (#8). During the further development of one of his own inventions, the coordinator developed an extensive network within Erasmus Medical Center and strong connections to the technical and legal divisions of the hospital. After realizing that other nurse innovators struggled with the same type of problems as he faced during the development of his invention, he started helping his co-workers and mobilized his network to advance the nurse innovations of others. At first, he mainly targeted colleagues in the Children’s Intensive Care unit and started venturing out to other departments soon thereafter. Especially in the early years, Create4Care was mainly a personal project. It was financed by temporal budgets/grants, operated as an informal network, and many actors contributed voluntarily. It took more than 5 years before permanent funding was obtained and Create4Care was officially recognized as a department of Erasmus Medical Center. As Create4Care grew in terms of size and level of professionalism, the interdependencies between ecosystem actors changed. In particular, creating a more professional nurse innovation development trajectory also made the process more complex and formalized. This can easily result in a situation in which the process becomes more ‘detached’ from the nurses that contribute the ideas/prototypes and provide feedback. To prevent this and to ensure that the nurses understand why certain steps are crucial, Create4Care has developed several nurse innovation training programs in recent years. These programs mainly target innovation literacy and range from short workshops to dedicated training programs (consisting of online and in-class elements). Educating the nurses in innovation enhances mutual understanding, increases their confidence to participate in the development of innovations, and ensures that the different parts of the ecosystem co-evolve.

##### 3.2.2.4. Balancing profit and non-profit

Because of the substantial development costs of most nurse innovations, recouping some of the investment costs via licensing agreements or other means may be tempting, and considered necessary. Yet, Create4Care recognized that a predominant focus on commercialization would be disadvantageous, and can derail diffusion. Except for the business managers (#1,2) and entrepreneur (#13), all interviewees indicated that “*enjoyment*” and/or “*contributing to a greater good*” was their main motivation for participating in Create4Care. Focusing only on nurse innovation with commercial potential and neglecting other types of innovations that bare significant use value is expected to drive out these intrinsic motivations (52, 53). Hence, within Create4Care’s culture, it is well-accepted that many innovations will never be commercialized, but can still be very meaningful when diffused freely to peers.

##### 3.2.2.5. Top management support

Importantly, freely sharing innovations certainly helped to accomplish diffusion, but for this diffusion pathway, top management support is indispensable. Many nurse innovations mainly generate indirect benefits [more effective treatments or a less physically demanding working environment, see (1)]. These benefits are difficult to quantify in economic terms (3), and investing in the development of nurse innovations without commercial potential is difficult without (financial) support from hospital management. Interestingly, we observed that top management support at Create4Care was *not* provided in advance. As mentioned, Create4Care emerged bottom-up as a result of the actions and efforts of a nurse innovator. He (#3) provided a proof of concept and, especially in the early years, asked for modest budgets that he knew they could not be refused (e.g., using schooling budgets to develop prototypes and produce initial test versions). As Create4Care grew, their approach became more professional, and, with the help of the manager of Create4Care (#4), the nurse innovator started formalizing and embedding Create4Care in the organizational chart. Only after obtaining a critical mass and showing numerous successful projects, permanent and larger budgets were asked for, and allocated. Top management was always supportive and considered it a low-risk investment, given the promising results that Create4Care’s contributors could demonstrate at the time. Hence, although supporting and investing in nurse innovations with limited or uncertain economic benefits is essential, top management support was not such that big budgets were allocated without convincing results. In contrast, as an evaluation of the Swedish Makerspace Program by Svensson and Hartmann (3) shows, top-down implementation of makerspace programs (with large budgets being assigned in advance) is unlikely to optimize the diffusion of nurse innovations.

## 4. Discussion

Our study shows that nurses face multiple barriers that keep them from innovating, from solving their personal problems, and from spreading their innovations to the benefit of all in particular. These barriers are unlikely to be fully removed via training and innovation support systems. Based on our case study, we provide a concrete example of how a medical makerspace, and innovation support systems in a broader sense, can be designed to more adequately address nurse innovation and (in particular) diffusion barriers. The two main elements of the practical solution that we identified are: (1) Support systems should facilitate that others may lead the development and diffusion of nurse innovations and (2) The support system should promote that actors with distinct expertise and skills (nursing, engineering, commercialization, legal) integrate their functional specializations within an innovation ecosystem. Below, we discuss the implications of these findings in greater detail.

### 4.1. Implications and contributions

Previous studies of nurse innovation have mainly focused on removing innovation barriers and tended to ignore subsequent diffusion to the benefit of other nurses. With regard to removing barriers, innovation training (1) or educational programs (8) improve opportunity recognition and may equip nurses with the necessary skills to conduct basic market research, develop their innovation, and pitch an innovation to potential investors [also see (53)]. Also, medical makerspaces provide access to the necessary equipment to develop prototypes and can connect nurse innovators to commercial businesses (3, 31). Yet, even when nurses have the right skills and are provided with technical support, innovation within the medical sector remains a lengthy and complex process and diffusion is not evident (11, 14). In addition, because most innovations are developed in reaction to practical problems (8, 12) and do not generate significant commercial value (14, 54), only nurses who are intrinsically motivated and enjoy the innovation process are expected to further develop and diffuse their innovations (46, 47, 52, 53).

Our case study shows that both the rate and diffusion of nurse innovations can be significantly improved if others take a leading role in the development and diffusion processes. These others often bring specialized knowledge, skills, and competencies that nurse innovators may lack. This significantly lowers the barrier for nurses to engage in innovation, speeds up the innovation process, and increases the likelihood of diffusion. In other words, external contributors help to bridge the gap between early innovation prototyping, and broad diffusion via commercial or peer-to-peer pathways. Crucially, our case illustrates that the involvement of others does not have to be at the expense of nurses’ involvement and that large groups of nurses can still be actively involved in the development process.

We make two contributions that are of theoretical importance. First, we contribute to understanding barriers in the nurse innovation–diffusion process from a more psychological point of view. In this view, barriers are not objective in that they can be fully removed via training/education and technical support systems. Instead, perceived barriers are both objective and subjective, and interact with one another within a complex system of personal and work-related goals (44, 46, 53). This has important implications for practice. For example, it implies that the likelihood of further developing and diffusing a nurse’s innovation is not only a function of ability and creativity but of factors such as age or hierarchical level. Older individuals, for example, may value personal over professional goals while individuals who just started their nursing career may erroneously lack confidence in their innovation abilities (46). Fully taking advantage of nurses’ innovation potential would necessitate that hospitals develop support systems in such a way that nurse innovators are not forced to take a leading role in the further development of the innovations.

Second, we identified that an ecosystem perspective on nurse innovation and diffusion is beneficial to develop better systems, that is, innovation systems in which diffusion occurs more often. The benefits of a more open approach to innovation (25–27) are currently gaining traction within the medical sector. An ecosystem perspective to open innovation highlights the importance of complementarity among a set of actors with diverse functional skills (49). Given the technological, legal, and market complexities of nurse innovations, it is unlikely that a single nurse possesses all the necessary skills to develop and diffuse her/himself, or has a network that can help with all relevant tasks. In an innovation ecosystem, individuals do not only maximize their own output but also that of others within a community setting. The conditions under which individuals show altruistic behavior and maximize the output of others are likely to be dependent on the goals and way the ecosystem is being managed. For example, a nurse innovation ecosystem that balances diffusion via commercial and peer-to-peer pathways and has no requirements to break-even is more likely to elicit altruistic behaviors among members of the ecosystem, compared to one with a focus on commercial revenues only. Similarly, an ecosystem that is developed bottom-up and is tailored to the needs and requirements of all members of the ecosystem would elicit higher levels of engagement and commitment among members. The critical success factors that we identified provide a starting point for further investigating and understanding the inner workings of such nurse innovation ecosystems and why these ecosystems are successful.

### 4.2. Practical implications and transferability of the results

Our study provides important insights for managers and practitioners that seek to open a medical makerspace (or fablab or living lab) to facilitate nurse innovation. Most crucially, our study shows that a one-size-fits-all approach to creating a medical makerspaces is unlikely to be successful. The founders of Create4Care have built their innovation ecosystem over a long period of time; initially at a very modest level with limited budgets, and lots of voluntary contributors from their emerging network. While doing so, they incorporated best practices developed elsewhere but carefully tailored these best practices to the local innovation requirements of nurse innovations at Erasmus Medical Center. In addition, they balanced the needs of different stakeholder groups (nurses, patients, the hospital, and commercial parties) in such a way that there was not one beneficiary, but that all groups equally benefited from their contributions to the ecosystem. Only later, they gradually expanded their activities and started asking for larger budgets, permanent lab facilities, and official organizational embeddedness. These findings imply that makerspaces that are created in response to corporate policies, that is, planned in top-down fashion, are likely to be less effective than those that emerge bottom-up. To managers and other decision-makers, it is recommended not to try to create a makerspace or similar support system for nurse innovation overnight. Instead, back up those employees who truly care about innovation and diffusion processes, and facilitate an emerging ecosystem.

Other important design factors for nurse innovation makerspaces directly follow from the critical success factors discussed in section 3.2.2. First, it is important to nurture a community feeling, by developing a shared purpose first: solving nurse innovation problems with practical solutions, from which any nurse can benefit—regardless of the most appropriate diffusion pathway (commercially, peer-to-peer, or both). Second, avoid the emerging ecosystem’s activities are derailed by excessive revenue targets, to be obtained from licensing or selling nurse innovations to commercial partners (which is the dominant mode of most technology transfer offices at hospitals). In our case study, this pitfall was avoided by recognizing the importance of nurses as a source of innovation, and by accepting some social responsibility for diffusing innovations even for free. Thirdly, top management support is indispensable for such a system to sustain in the longer run, if only because part of the makerspace’s expenditures have to be covered by lumpsum budgets. Recall, however, that top managers at Erasmus Medical Center never felt that budget requests from Create4Care’s initiators were unrealistic, because viable and generally useful innovations could be demonstrated first—the recommended gradual process of bottom-up emergence secured that all investments were considered low-risk.

### 4.3. Limitations and future research

Our study had limitations that translate directly into recommendations for continued research. First, although our findings mainly emphasize the importance of “customizing” makerspaces to the needs of stakeholders, the relationships that we identified may not fully transfer to other settings or even other hospitals. Erasmus Medical Center is a research-intensive environment with a proven infrastructure for the development and diffusion of physician-led innovations. Create4Care makes use of this infrastructure (e.g., legal and technical expertise). Even though this is a representative setting for academic hospitals in the Netherlands, it is not necessarily representative of all academic hospitals and peripheral hospitals in particular. This creates the need to study the effectiveness and inner workings of nurse innovation ecosystems in a variety of settings and countries.

Second, we did not design this study to uncover individual-level decision-making processes. Instead, as is common in qualitative research, the anticipatory mechanisms that we identified surfaced as the result of our combination of deductive and inductive reasoning (39–41). Future studies should follow up on these findings and such research may want to make use of experimental designs to test the causal relationships that we propose.

Third, in terms of the selection of the interviewees, we relied on our on-site observations of the innovation and diffusion processes to select key informants. This is both a strength and a limitation. Follow-up studies may consider including a broader range of stakeholders that are involved in the nurse innovation–diffusion processes such as patients, patient organizations, commercial parties, innovation adopters, and top-level managers.

Finally, our study provides a starting point for understanding how successful nurse innovation ecosystems work, but the interrelations between the different critical success factors should be investigated in future work. These interrelations are also likely to change with the advent of artificial intelligence (AI) and tools such as ChatGPT and Bing Chat becoming available to a wide audience. Such tools empower nurses to develop different types of nurse innovations and the successful diffusion of AI-powered nurse innovations via peer-to-peer or commercial pathways likely requires different types of competencies and external relationships.

## Data availability statement

The datasets presented in this article are not readily available due to the nature of the research. As is common in qualitative research and to ensure that the participants could speak freely, we informed the interviewees that the data would not be shared with company management or other third parties. The participants did not give written consent for their data to be shared publicly. Requests to access the datasets should be directed to CR, j.p.c.rigtering@uu.nl.

## Ethics statement

The studies involving human participants were reviewed and approved by Ethics Committee Faculty of Law, Economics, and Governance of Utrecht University. Written informed consent for participation was not required for this study in accordance with the national legislation and the institutional requirements.

## Author contributions

CR: conceptualization, methodology, formal analysis, investigation, writing—original draft, writing—review and editing, and visualization. LS: conceptualization, formal analysis, validation, investigation, and writing—review and editing. JJ: investigation, formal analysis, writing—review and editing, supervision, and project administration. All authors contributed to the article and approved the submitted version.

## Conflict of interest

The authors declare that the research was conducted in the absence of any commercial or financial relationships that could be construed as a potential conflict of interest.

## Publisher’s note

All claims expressed in this article are solely those of the authors and do not necessarily represent those of their affiliated organizations, or those of the publisher, the editors and the reviewers. Any product that may be evaluated in this article, or claim that may be made by its manufacturer, is not guaranteed or endorsed by the publisher.

## Footnotes

^1^Von Hippel et al. (14) show that, across a large range of studies, diffusion rates to not exceed 25%. In comparison, more than 46% of the nurse innovations developed at Create4Care diffused to a broader audience.

^2^Class I medical devices are devices that have a low to moderate risk to the patient and/or user. Higher classes (Class II or III) medical devices have moderate to high risks to the patient.

## References

[1] Gao L Lu Q Hou X Ou J Wang M. Effectiveness of a nursing innovation workshop at enhancing nurses’ innovation abilities: a quasi-experimental study. Nurs Open. (2022) 9:418–27. doi: 10.1002/nop2.1080, PMID: 34687153 PMC8685873

[2] Leary M Villarruel AM Richmond TS. Creating an innovation infrastructure in academic nursing. J Prof Nurs. (2022) 38:83–8. doi: 10.1016/j.profnurs.2021.12.005, PMID: 35042594

[3] Svensson PO Hartmann RK. Policies to promote user innovation: makerspaces and clinician innovation in Swedish hospitals. Res Policy. (2018) 47:277–88. doi: 10.1016/j.respol.2017.11.006

[4] National Academy of Medicine. The future of nursing 2020–2030: Charting a path to achieve health equity. Washington, DC: The National Academies Press (2021). 34524769

[5] Clark E Webster-Henderson B. Innovation and its contribution to the scholarship of learning and teaching. Nurse Educ Today. (2012) 32:729–31. doi: 10.1016/j.nedt.2012.06.001 22749245

[6] Roddy L Polfuss M. Employing design thinking methods in nursing to improve patient outcomes. Nurs Forum. (2020) 55:553–8. doi: 10.1111/nuf.12461, PMID: 32497281

[7] Shahsavari Isfahani S Hosseini MA Fallahi Khoshknab M Peyrovi H Khanke HR. Nurses’ creativity: advantage or disadvantage. Iran Red Crescent Med J. (2015) 17:e20895. doi: 10.5812/ircmj.20895, PMID: 25793116 PMC4353217

[8] O'Hara S Ackerman MH Raderstorf T Kilbridge JF Melnyk BM. Building and sustaining a culture of innovation in nursing academics, research, policy, and practice: outcomes of the National Innovation Summit. J Prof Nurs. (2022) 43:5–11. doi: 10.1016/j.profnurs.2022.08.001, PMID: 36496244

[9] Grant E.J. ‘Resources and recognition for advancing nurses’ ideas and ingenuity’. American Nurses Association. (2022). Available at: https://www.myamericannurse.com/ana-innovation-central/ (Accessed 3 January 2023).

[10] Kichgessner JC Keeling AW. Nursing rural America: Perspectives from the early 20th century. New York, N.Y: Springer (2015).

[11] Berwick DM. Disseminating innovations in health care. J Am Med Assoc. (2003) 289:1969–75. doi: 10.1001/jama.289.15.1969 12697800

[12] Debono DS Greenfield D Travaglia JF Long JC Black D Johnson J. Nurses’ workarounds in acute healthcare settings: a scoping review. BMC Health Serv Res. (2013) 13:175. doi: 10.1186/1472-6963-13-175, PMID: 23663305 PMC3663687

[13] White KR Pillay R Huang X. Nurse leaders and the innovation competence gap. Nurs Outlook. (2016) 64:255–61. doi: 10.1016/j.outlook.2015.12.007 26827191

[14] von Hippel E DeMonaco H de Jong JPJ. Market failure in the diffusion of clinician-developed innovations: the case of off-label drug discoveries. Sci Public Policy. (2017) 44:121–31. doi: 10.1093/scipol/scw042

[15] Davis CR Glasgow MES. Nurse-scientists and nurse-engineers. Am Nurse Today. (2017). Available at: https://www.myamericannurse.com/nurse-scientists-nurse-engineers/

[16] Nelson R. Nusing innovation. Am J Nurs. (2020) 120:18–9. doi: 10.1097/01.NAJ.0000656300.77328.fe, PMID: 32079789

[17] Melnyk BM. The current research to evidence-based practice time gap is now 15 instead of 17 years: urgent action is needed. Wolrdviews Evid Based Nurs. (2021) 18:318–9. doi: 10.1111/wvn.12546 34799980

[18] Nählinder J. Where are all the female innovators? Nurses as innovators in a public sector innovation project. J Technol Manag Innov. (2010) 5:14–29. doi: 10.4067/S0718-27242010000100002

[19] Giuliano KK Landsman K. A nurse innovator paves the way for other nurses. Am J Nurs. (2022) 122:59–61. doi: 10.1097/01.NAJ.0000904116.15025.72, PMID: 36384801

[20] Glasgow MES Colbert A Viator J. The nurse-engineer: a new role to improve nurse technology interface and patient care device innovations. J Nurs Scholarsh. (2018) 50:601–11. doi: 10.1111/jnu.12431, PMID: 30221824

[21] Boore J Porter S. Education for entrepreneurship in nursing. Nurse Educ Today. (2011) 31:184–91. doi: 10.1016/j.nedt.2010.05.016 20594624

[22] Barr TL Malloch K Ackerman MH Raderstorf T Melnyk BM. A blueprint for nursing innovation centers. Nurs Outlook. (2021) 69:969–81. doi: 10.1016/j.outlook.2021.05.006, PMID: 34183188 PMC9077284

[23] Felton G. The dean, faculty research, and entrepreneurship. J Prof Nurs. (1986) 2:80–133. doi: 10.1016/S8755-7223(86)80072-2, PMID: 3633935

[24] Giuliano KK Sup FC Benjamin E Krishnamurty S. Innovate: preparing nurses to be health care innovation leaders. Nurs Adm Q. (2022) 46:255–65. doi: 10.1097/NAQ.0000000000000529, PMID: 35639532 PMC9162070

[25] Buur J Matthews B. Participatory innovation. Int J Innov Manag. (2008) 12:255–73. doi: 10.1142/S1363919608001996

[26] Chesbrough HW. Open innovation: The new imperative for creating and profiting from technology. Boston, MA: Harvard Business School Press (2003).

[27] Eftekhari N Bogers M. Open for entrepreneurship: how open innovation can foster new venture creation. Creat Innov Manag. (2015) 24:574–84. doi: 10.1111/caim.12136

[28] Fabfoundation.org ‘Getting started with fab labs’. (2023). Available at: https://fabfoundation.org/getting-started/ (Accessed 19 June 2023).

[29] Lab 4 Living ‘Lab4Living: Design for life and for living.’(2023). Available at: https://lab4living.org.uk/ (Accessed 19 June 2023).

[30] Makernurse.com Makernurse.Com, MakerNurse: Powered by MakerHealth. (2022). Available at: http://makernurse.com/.

[31] Marshall DR McGrew DA. Creativity and innovation in health care: opening a hospital makerspace. Nurse Lead. (2017) 15:56–8. doi: 10.1016/j.mnl.2016.10.002

[32] Yin RK. Case study research: Design and methods. 4th ed. Thousand Oaks, CA: Sage (2009).

[33] de Jong JPJ von Hippel E. Transfers of user process innovations to producers: a study of Dutch high tech firms. Res Policy. (2009) 38:1181–91. doi: 10.1016/j.respol.2009.04.005

[34] Kumar N Stern LW Anderson JC. Conducting interorganizational research using key informants. Acad Manag J. (1993) 36:1633–51. doi: 10.2307/256824

[35] Fisher RP Ross SJ Cahill BS. Interviewing witnesses and victims In: Granhag PA, editor. Foundations and trends in entrepreneurship. New York, NY: Routledge (2013)

[36] Tulving E. Episodic memory: from mind to brain. Annu Rev Psychol. (2002) 53:1–25. doi: 10.1146/annurev.psych.53.100901.135114 11752477

[37] Gioia DA Corley KG Hamilton AL. Seeking qualitative rigor in inductive research: notes on the Gioia methodology. Organ Res Methods. (2012) 16:15–31. doi: 10.1177/1094428112452151

[38] Strauss A Corbin J. Grounded theory in practice. Thousand Oaks, CA: Sage (1997).

[39] Stake RE. The art of case study research. Thousand Oaks, CA: Sage (1995).

[40] Suddaby R. From the editors: what grounded theory is not. Acad Manag J. (2006) 49:633–42. doi: 10.5465/amj.2006.22083020

[41] Fereday J Muir-Cochrane E. Demonstrating rigor using thematic analysis: a hybrid approach of inductive and deductive coding and theme development. Int J Qual Methods. (2006) 5:80–92. doi: 10.1177/160940690600500107

[42] Rogers EM. Diffusion of innovation. New York: Free Press (1962).

[43] von Hippel E. Free innovation. Cambridge, MA: MIT Press (2017).

[44] Bandura A. Self-efficacy: towards a unifying theory of behavioral change. Psychol Rev. (1977) 84:191–215. doi: 10.1037/0033-295X.84.2.191 847061

[45] Maxwell AL Jeffrey SA Lévesque M. Business angel early stage decision making. J Bus Ventur. (2011) 26:212–25. doi: 10.1016/j.jbusvent.2009.09.002

[46] Bandura A. Social cognitive theory of self-regulation. Organ Behav Hum Decis Process. (1991) 50:248–87. doi: 10.1016/0749-5978(91)90022-L

[47] Bandura A. Human agency in social cognitive theory. Am Psychol. (1989) 44:1175–84. doi: 10.1037/0003-066X.44.9.1175 2782727

[48] Michaelis TL Scheaf DJ Carr JC Pollack JM. An agentic perspective of resourcefulness: self-reliant and joint resourcefulness behaviors within the entrepreneurship process. J Bus Ventur. (2022) 37:106083. doi: 10.1016/j.jbusvent.2020.106083

[49] Stam E Van de Ven A. Entrepreneurial ecosystem elements. Small Bus Econ. (2021) 56:809–32. doi: 10.1007/s11187-019-00270-6

[50] Nambisan S Baron RA. Entrepreneurship in innovation ecosystems: entrepreneurs’ self-regulatory processes and their implications for new venture success. Entrep Theory Pract. (2013) 37:1071–97. doi: 10.1111/j.1540-6520.2012.00519.x

[51] Astley WG. The two ecologies: population and community perspectives on organizational evolution. Adm Sci Q. (1985) 30:224–41. doi: 10.2307/2393106

[52] Ryan RM Deci EL. Intrinsic and extrinsic motivations: classical definitions and new direction. Contemp Educ Psychol. (2000) 25:54–67. doi: 10.1006/ceps.1999.1020, PMID: 10620381

[53] Ryan RM Deci EL. Self-determindation theory: Basic psychological needs in motivation, development, and wellness. New York, NY: The Guilford Press (2017).

[54] de Jong JP Hippel AC Gault F Kuusisto J Raasch C. Market failure in the diffusion of consumer-developed innovations: patterns in Finland. Res Policy. (2015) 44:1856–65. doi: 10.1016/j.respol.2015.06.015

