# Supplementary material for: How to bridge the nurse innovation–diffusion gap? An in-depth case study of Create4Care
Source: Front Public Health. 2023 Aug 3;11:1209965. doi: 10.3389/fpubh.2023.1209965 (PMC10434511; doi:10.3389/fpubh.2023.1209965)
Supplement: Supplementary file 1 [file Table_1.DOCX]

Supplementary Material

How to bridge the nurse innovation–diffusion gap? An in-depth case study of Create4Care

Coen Rigtering^*^, Lara Spaans, Jeroen de Jong

*** Correspondence:**Corresponding Author: Coen Rigtering, [j.p.c.rigtering@uu.nl](mailto:j.p.c.rigtering@uu.nl)

# Supplementary materials A: Interview guide

**The diffusion of innovation**

**Interview checklist EramusMC Create4Care**

Respondent:

Interviewer(s):

Date:

*NOTES*

*Normal. Question to be asked*

- *Bullet . Potential follow-up question – will usually NOT be asked unless the previous answer counsels it*

**Start of the interview** (2-3 minutes)

Personal introduction

Introduction research: We conduct research on Create4Care's innovation projects. We focus on broader dissemination, from the moment a working prototype is available.

Explain data handling, data management, and privacy.

**1. Involvement in Create4Care and projects** (10 minutes max)

1a. What is your work/task?

1b. What do you find attractive in your regular work/task? Why did you choose this work?

1c. How are you involved with Create4Care? What part of your time?

1d. What projects have you been involved in?

- Provide the list of completed projects (if necessary).

1e. How are you usually involved in projects? In what role?

- - ..initiator/idea generator/problem solver
- ..developer/designer
- ..coach/supervisor
- ..knowledge, expertise, expert promoter
- ..influencer, resources/budget, power promoter
- ..relationships, networking, relationship promoter
- ..processes, knowledge of the organization, process promoter
- opponent, devil's advocate
- ..feedback/test/pilot
- ..disseminator/change agent)

**2. Activities - focused on project dissemination** (10-20 minutes)

At some point a working prototype is available. Then the question is whether the innovation can spread: to other users inside or outside ErasmusMC. This can be done directly between users, through a producer, or by starting a new company.

2a. What concrete actions are you taking to spread innovations?

- Can you mention some concrete examples
- Do missing activities come to mind?
- Refer to the projects that the respondent is involved in: do you recall any activities?

2b. In your opinion, what are the main barriers to project dissemination in general?

- Were there specific barriers within projects? How do you deal with them?

**3. Activities - focused on environment and infrastructure** (10-15 minutes)

In addition to the projects, we want to address the preconditions and conditions for dissemination.

3a. Do you ever do things targeting Create4care's resources/facilities yourself?

3b. Do you ever do things focused on the conditions for dissemination?

- - Do any missing activities come to mind?

**4. Motives** (10-15 minutes)

4a. Why do you put effort into Create4care's projects? Motives?

4b. Why specifically do you make efforts to disseminate projects?

- - What are your most important motives? Which ones are less important? Which are irrelevant?

4c. Why do you make efforts on some projects and not others?

**5. Other Characteristics** (5-10 minutes)

5a. Can you tell me a little bit more about your background?

- - Education?
  - Work experience? What work for current position?
  - Position in the hospital? Network connections? With which department/people?
  - Experience with management/leadership?
  - Experience with commerce/entrepreneurship?
  - Technical/development experience? Use experience (regarding projects)?
  - Experience with dissemination initiatives/communication?

5b. Time investment Create4care? Also free time?

**6. Most important diffusion actors within Create4Care** (2 minutes)

6a. Who contributes most to the diffusion of innovations within Create4Care?

**7. Final**

Many thanks for participating in the interview!

7a Provide the respondent with the opportunity to ask questions.

# 
